# The Trimeric Autotransporter Adhesin SadA from *Salmonella* spp. as a Novel Bacterial Surface Display System

**DOI:** 10.3390/vaccines12040399

**Published:** 2024-04-09

**Authors:** Shuli Sang, Wenge Song, Lu Lu, Qikun Ou, Yiyan Guan, Haoxia Tao, Yanchun Wang, Chunjie Liu

**Affiliations:** 1 State Key Laboratory of Pathogen and Biosecurity, Institute of Biotechnology, Academy of Military Medical Sciences, 20 Dongda Street, Fengtai District, Beijing 100071, China; sangshuli@bmi.ac.cn (S.S.); wgsongzq@163.com (W.S.); lulu52102022@163.com (L.L.); 18877961233@163.com (Q.O.); yiyiyiyan610@163.com (Y.G.); taohaoxia@126.com (H.T.); 2 School of Basic Medical Sciences, Guangxi Medical University, 22 Shuangyong Road, Nanning 530021, China

**Keywords:** trimeric autotransporter adhesins, SadA, bacterial surface display, *Salmonella* spp.

## Abstract

Bacterial surface display platforms have been developed for applications such as vaccine delivery and peptide library screening. The type V secretion system is an attractive anchoring motif for the surface expression of foreign proteins in gram-negative bacteria. SadA belongs to subtype C of the type V secretion system derived from *Salmonella* spp. and promotes biofilm formation and host cell adherence. The inner membrane lipoprotein SadB is important for SadA translocation. In this study, SadA was used as an anchoring motif to expose heterologous proteins in *Salmonella typhimurium* using SadB. The ability of SadA to display heterologous proteins on the *S. typhimurium* surface in the presence of SadB was approximately three-fold higher than that in its absence of SadB. Compared to full-length SadA, truncated SadAs (SadA^877^ and SadA^269^) showed similar display capacities when exposing the B-cell epitopes of urease B from *Helicobacter pylori* (UreB158–172aa and UreB349–363aa). We grafted different protein domains, including mScarlet (red fluorescent protein), the urease B fragment (UreBm) from *H. pylori* SS1, and/or protective antigen domain 4 from *Bacillus anthracis* A16R (PAD4), onto SadA^877^ or SadA^1292^. Whole-cell dot blotting, immunofluorescence, and flow cytometric analyses confirmed the localization of Flag×3-mScarlet (~30 kDa) and Flag×3-UreBm-mScarlet (~58 kDa) to the *S. typhimurium* surface using truncated SadA^877^ or SadA^1292^ as an anchoring motif. However, Flag×3-UreBm-PAD4-mScarlet (~75 kDa) was displayed on *S. typhimurium* using SadA^1292^. The oral administrated pSadBA^1292^-FUM/StmΔ*ygeA*Δ*murI* and pSadBA^877^-FUM/StmΔ*ygeA*Δ*murI* could elicit a significant mucosal and humoral immunity response. SadA could thus be used as an anchoring motif for the surface expression of large heterologous proteins as a potential strategy for attenuated bacterial vaccine development.

## 1. Introduction

Bacterial cell surface display allows for the production of target biomolecules, such as peptides or proteins on the bacterial surface. This technology has been investigated and developed for several applications, including peptide library screening and whole-cell biocatalysts, especially for vaccine delivery with improvements in immune effectiveness. Surface display vaccines can increase the immunogenicity of foreign antigens by facilitating their recognition by the immune system. For example, the surface display of antigens derived from *Mycobacterium tuberculosis* on *Salmonella typhimurium* [1] or *Lactiplantibacillus plantarum* [2] evokes a stronger immune response compared to that with cytoplasmic delivery. Surface display vaccines also have advantages such as their ease of use and low manufacturing costs.

A common strategy used in surface display systems is to fuse a protein or peptide with an anchoring motif that is important for the stability of heterologous protein expression onto the bacterial surface. Various proteins, including fimbria proteins [3], outer membrane proteins [4,5] (OmpA, OmpT, OmpC, MipA), ice-nucleation protein (INP) [6], and monomeric autotransporters (the adhesin involved in diffuse adherence (AIDA-I), hemoglobulin-binding protease (Hbp), MisL, ShdA) [7,8,9,10], have been developed as anchoring motifs in gram-negative bacterial display systems. However, each anchoring motif has different drawbacks, such as size limitation, mechanical fragility, and steric hindrance.

Trimeric autotransporter adhesins (TAAs) are type V secretion system subtype C (T5cSS) proteins, which are involved in several aspects of the infection process in gram-negative bacteria and developed as candidates for recombinant subunit vaccines [11]. TAAs are composed of three identical polypeptide chains and share a common N-terminus–head–neck–stalk–membrane anchor–C-terminus architecture, varying greatly in length, from 23 nm [12] to 240 nm [13]. Nakatani reported the trimeric autotransporter adhesin (AtaA) from *Acinetobacter* sp. Tol 5 could be used to display a His-tag in *Escherichia coli* cells. This on-fiber display system could change the distance between the cell surface and the displayed biomolecule [14]. Phan et al. used the truncated mutant of trimeric autotransporter UpaG in uropathogenic *E. coli* to display calmodulin and a nanobody binding to a green fluorescent protein on *E. coli* cells with the overexpression of the BAM (β-barrel–assembly machinery) complex [15]. The length, trimeric form, and autodisplay characteristics indicate that TAAs could serve as display platforms for efficient vaccine development.

Attenuated *Salmonella* spp. have been developed to deliver heterologous antigens from viruses, bacteria, protozoans, and fungi to induce immune responses against pathogenic infections [3,7,16,17]. SadA, the TAA that has been characterized in *S. typhimurium*, consists of polypeptides comprising 1461 amino acids and forms a 108 nm long model [18,19], whereas SadB, a small inner membrane lipoprotein in *Salmonella* spp., is important for SadA translocation. Grin et al. reported a significantly higher immunofluorescence signal level in a P_BAD_::*sadBA Salmonella enterica* strain than in a P_BAD_::*sadA S. enterica* strain upon L-arabinose addition, suggesting the direct involvement of SadB in the biogenesis of SadA [20]. In this study, we first utilized full-length and truncated SadAs to display epitope peptides on the surface of *S. typhimurium* with the assistance of SadB. Furthermore, truncated SadAs were employed to display heterologous proteins of different sizes to test the passenger protein capacity. We evaluated the immunogenicity of the heterologous antigen surface displayed on *S. typhimurium* using SadA as an anchoring motif in a mouse model. Our work indicates that SadA from *S. typhimurium* could be developed into a novel antigen surface expression system for studying attenuated live vector vaccines.

## 2. Materials and Methods

### 2.1. Plasmid Construction

The primers and plasmids used in this study are listed in Tables S1 and S2. The *sadA*^1–168^*-flag×*3*-sadA*^169–990^ and *sadA*^1–168^*-flag×*3*-a3c10-a1h10-sadA*^169–990^ fragments were synthesized commercially and subcloned into pUC57 (General Biol, Anhui, China), as shown in Table S3. The sequence of the *sadA*^331–1462^ fragment with restriction enzyme sites (*NheI* and *HindIII*) was amplified via PCR using *S. typhimurium* 1.1174 genomic DNA as the template. The *sadA*^991–4386^ gene (5′-*NheI*–3′-*HindIII* restriction fragment) and the *sadA*^1–168^*-flag×3-sadA*^169–990^ fragment (with 5′-*NcoI*–3′-*NheI* restriction enzyme sites) amplified from *pUC57-sadA*^1–168^*-flag×*3*-sadA*^169–990^ were subcloned into the pTrc99A vector, which was named pSadA-Flag×3. The *sadB* gene with a 5′-EcoRI site and the *sadA*^1–168^*-flag×*3*-sadA*^169–990^ fragment with a 3′-*HindIII* site were amplified via PCR using *S. typhimurium* and pSadA-Flag×3 as the templates, respectively. DNA fragments of *sadB-sadA*^1–168^*-flag×3-sadA^169–4386^* with *EcoRI* and *NheI* sites were amplified via overlap extension PCR. To construct pSadBA-Flag×3, the *sadB-sadA*^1–168^*-flag×*3*-sadA*^169–4386^ (5′-*EcoRI*–3′-*HindIII* restriction fragment) was ligated into the pTrc99A vector digested with *EcoRI* and *HindIII*. The genes of *sadBA* derivatives fused with the genes *flag×*3, *ureB*158–172aa, and *ureB*349–363aa from *H. pylori* SS1 were constructed via overlap extension PCR using the primers described in Table S2. The genes of sadBA derivatives were ligated into *EcoRI*/*HindIII*-digested pTrc99A. The genes of *ureBm*, *pad4*, and *mScarlet* were amplified via PCR using *H. pylori* SS1 genomic DNA, *B. anthracis* A16R genomic DNA, and the plasmid pJOE-mScarlet as templates, respectively. *sadBA*^877^*-FM* (*flag×*3*-mScarlet*), *sadBA*^877^*-FUM* (*flag×*3*-ureBm-mScarlet*), *sadBA*^877^*-FUPM* (*flag×*3*-ureBm-pad4-mScarlet*), *sadBA*^1292^*-FM*, *sadBA*^1292^*-FUM*, and *sadBA*^1292^*-FUPM* were obtained using overlap extension PCR with *EcoRI*/*HindIII* restriction sites (Table S2). These genes were ligated into the same restriction sites in the pTrc99A vector. The plasmid for the intracellular production of FM (Flag×3-mScarlet) was amplified using the primer pairs shown in Table S1. The resulting PCR product was digested with *EcoRI*/*HindIII* and ligated into the same restriction sites of the pTrc99A vector to yield pFM. All the constructed recombinant plasmids were confirmed via sequencing (Tianyi Huiyuan, Beijing, China).

### 2.2. Chromosomal Deletion of sadA, ygeA, and murI Genes from S. typhimurium

The *S. typhimurium* 1.1174 parental strain was used for the construction of the StmΔ*sadA* strain. All genetic manipulations were performed using the CRISPR-Cas9 system [21]. Briefly, at an optical density (OD)_600_ = 0.6~0.7, bacterial cultures were placed on ice for 30 min and centrifugated at 3500× *g* for 8 min. After washing with ice-cold ddH_2_O, *S. typhimurium* cells were washed three times with 10% glycerol to make electrocompetent cells. Then, pCas was transformed into *S. typhimurium* cells. The N_20_ sequence was designed using the online design tool (https://sg.idtdna.com/site/order/designtool/index/CRISPR_CUSTOM, accessed on 9 March 2022), and inserted into pTargetF to obtain recombinant plasmid named pTargetF-*sadA*. Next, 517 bp upstream and 493 bp downstream sequences of the targeted region were cloned to obtain donor DNA using the primers shown in Table S2. The pCas/*S. typhimurium* was made into electrocompetent cells by adding arabinose at a final concentration of 10 mM for λ-Red induction. Then, 100 ng of pTargetF-*sadA* and 1 μg of donor DNA were co-transformed into fleshly grown electrocompetent pCas/*S. typhimurium* cells. The *sadA*-deleted clones were verified using PCR and DNA sequencing after overnight culture on Luria–Bertani (LB) plates containing kanamycin and spectinomycin (Table S2). The correct edited clone was cultured in LB with kanamycin and isopropyl-β-D-thiogalactopyranosid (IPTG) overnight at 30 °C to remove pTargetF. Subsequently, pCas was removed by culturing the strains overnight at 42 °C in LB medium without any antibiotics.

*ygeA* and *murI* genes were deleted from the chromosomal of the *S. typhimurium* 1.1174 parental strain according to the method mentioned above.

### 2.3. Strains and Growth Conditions

*Escherichia coli* strain DH5α used for cloning, and StmΔ*sadA* used for expression, were routinely cultured in LB broth with shaking or on agar supplemented with 100 μg/mL ampicillin and/or IPTG, where appropriate, at 37 °C. *S. typhimurium*, StmΔ*sadA*, and *B. anthracis* A16R were grown in LB broth at 37 °C for DNA isolation. The Δ*ygeA* and Δ*murI* double-deleted strain (StmΔ*ygeA*Δ*murI*) was cultured in LB broth supplemented with 100 μg/mL ampicillin and 5 mM D-glutamic acid (D-Glu) at 37 °C. *H. pylori* SS1 was cultured on Campylobacter Ager Base plates (CDRC, Shanghai, China) containing 7% fetal bovine serum in a microaerophilic atmosphere (80% N_2_, 5% O_2_, 15% CO_2_) for 3 days at 37 °C to extract genomic DNA.

### 2.4. Protein Expression and Analysis

StmΔ*sadA* and StmΔ*ygeA*Δ*murI* strains with the recombinant plasmid were grown aerobically in 5 mL of LB liquid medium (200 μg/mL ampicillin) and LB liquid medium (200 μg/mL ampicillin and 5 mM D-Glu) overnight at 37 °C, respectively. The next day, the cells were inoculated and induced with the addition of 1 mM IPTG when the OD_600_ value reached about 0.6, and then incubated for 15 h at 16 °C. 1 OD_600_ bacterial cells were harvested, washed twice with PBS, and resuspended in 200 μL of SDS sample buffer. A 30 μL sample was subjected to 4~12% SDS-PAGE (GenScript, Nanjing, China). The transferred PVDF membrane was blocked in PBST containing 5% skim milk at 4 °C overnight. Subsequently, the membrane was incubated with anti-Flag tag rabbit polyclonal antibody (diluted to 1:3000 with 5% skim milk in PBST; Easybio, Beijing, China), or A1H10 or A3C10 (1:2000 dilution) for 1 h at 37 °C. After three washes with PBST, the membrane was incubated with HRP-conjugated goat anti-rabbit IgG (1:5000 dilution; Easybio, Beijing, China) or HRP-conjugated goat anti-mouse IgG (1:5000 dilution; Abcam, Cambridge, UK) for 1 h at 37 °C. An ECL-enhanced Western Blot Analysis Kit (Easybio, Beijing, China) was used to detect the binding reactions.

### 2.5. Proteinase K Treatment of Bacterial Cells

Proteinase K digestion of the surface-exposed domains was performed as described by Xu et al. [6]. Briefly, the 1 OD_600_-induced bacterial cells were incubated with proteinase K at the final concentration of 400 μg/mL at 37 °C, and then we added 2 mM PMSF to stop the reaction. Aliquots were washed three times with PBS before protein electrophoresis, and immunofluorescence analyses were performed.

### 2.6. Immunofluorescence and Flow Cytometric Analyses

Immunofluorescence analysis was performed as described previously. Briefly, 1 OD_600_ cell was immobilized with 4% paraformaldehyde and blocked in PBS containing 1% bovine serum albumin (BSA) for 30 min. A rabbit anti-Flag antibody (diluted to 1:50 with 1% BSA in PBS) and an Alexa Fluor 488 goat anti-rabbit IgG antibody (1:50 dilution; Easybio, Beijing, China) were used for immunostaining. After washing with PBS, the cells were resuspended in 1 mL of PBS and transferred to a microplate (Nunc MicroWell 96, Thermo Fisher Scientific, New York, NY, USA). A SpectraMax i3× microplate reader was used to measure the fluorescence intensity using wavelengths of E_x_: 490 nm and E_m_: 535 nm. The amount of recombinant proteins displayed on the cell surface was quantified by dividing the fluorescence intensity of each sample by its OD_600_ value. Finally, the cells were mounted on poly-l-lysine-coated coverslips and dyed with DAPI, and immunofluorescence images were captured with a Nikon Ti2 inverted fluorescence microscope.

Flow cytometric analysis of the samples was performed. Each sample was labeled as described previously herein, using a rabbit anti-Flag antibody as the primary antibody (1:50 dilution) and Alexa Fluor 488 goat anti-rabbit IgG antibody (1:50 dilution) as the secondary antibody. Surface expression levels were evaluated by measuring fluorescence using a Northern Lights-CLC flow cytometer (CYTEK, Shanghai, China).

### 2.7. Whole-Cell Dot Blot

Recombinant *S. typhimurium* was immobilized in 4% paraformaldehyde and 0.04 OD_600_ cells were dropped onto the nitrocellulose membrane (NC). After drying for 30 min at 37 °C, the dot blot protocol proceeded as described previously herein using the rabbit anti-Flag-tag antibody and A3C10 as the primary antibody. As the whole-cell dot blot did not destroy the cell membrane and the cell membrane remained intact, the results showed whether the recombinant protein was displayed on the surface of the bacteria.

### 2.8. Immunization, Sample Collection, and Specific Antibody Detection by ELISA

For cultivation of pSadBA^1292^-FUM/StmΔ*ygeA*Δ*murI* and pSadBA^877^-FUM/StmΔ*ygeA*Δ*murI* strains, LB liquid medium was supplemented with 200 μg/mL ampicillin and 5 mM D-Glu. After inducing by 1 mM IPTG overnight at 16 °C, the cells were obtained by centrifugation and washed with PBS twice.

Three groups of 6-to-8-week-old BALB/c female mice (N = 10/group) were purchased from the Vital River Laboratory (Beijing, China) and immunized three times on days 0, 10, and 25. Group A and group B received oral pSadBA^1292^-FUM/StmΔ*ygeA*Δ*murI* and pSadBA^877^-FUM/StmΔ*ygeA*Δ*murI* with 10^9^ CFU for all three immunizations, respectively. Group C received oral PBS for all three immunizations as the control group. On day 31, sera and fecal samples from all mice were collected for the detection of antigen-specific IgG and secretory IgA (sIgA) levels. The fecal samples were treated according to the method reported by Zhang et al. [22].

Antigen-specific IgG levels in the serum and sIgA levels in the fecal samples were determined by ELISA according to the protocol published previously [22].

### 2.9. Statistical Analysis

All data were analyzed using GraphPad Prism 8.0.2 and are presented as the mean ± standard deviation. One-way ANOVA was employed to determine significant differences, and a *p*-value < 0.05 was considered statistically significant.

## 3. Results

### 3.1. Full-Length SadA Can Be Displayed on the Surface of S. typhimurium

To evaluate the suitability of SadA protein for surface display, a *sadA*-deleted mutant strain of *S. typhimurium* was constructed using the CRISPR-Cas9 genome editing system and selected as the expression host bacterium. The *sadA*-deleted mutant strain was confirmed by PCR and sequencing (Figure S1A).

**Figure 1:**
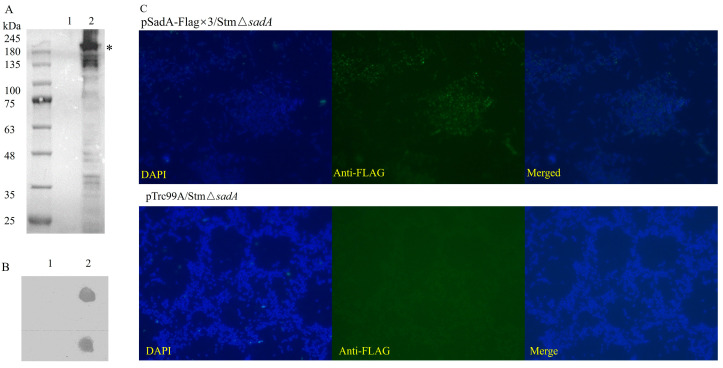
Displaying of the full-length SadA on the surface of *S. typhimurium* mutant cells. (**A**) Western blot of whole cell lysates to analyze expression of SadA in StmΔ*sadA*. The Flag tag was inserted at the N-terminus of SadA passenger. The recombinant protein bands were detected by incubating the PVDF using anti-Flag-tag antibody. The putative position of monomeric form (*) was indicated on the right side of the panel. Lane1, pTrc99A/StmΔ*sadA*; lane 2, pSadA-Flag×3/StmΔ*sadA*. Dot blot of whole cells showed the surface display of Flag-tagged SadA on StmΔ*sadA* (**B**). Lane1, pTrc99A/StmΔ*sadA*; lane2, pSadA-Flag×3/StmΔ*sadA*. (**C**) Cell surface display of Flag-tagged SadA by immunofluorescence staining using anti-Flag-tag primary antibody and AlexaFluor 488 conjugated secondary antibody (Objective, 100×; Magnification, 1000×).

Then, we constructed a Flag×3-tagged full-length SadA to confirm its surface display on StmΔ*sadA* cells. The recombinant protein was expressed in StmΔ*sadA* and the Flag-tag display on the surface was tested. We confirmed the production of the Flag-tagged SadA in StmΔ*sadA* through immunoblotting using the anti-Flag-tag antibody. The expressed protein was detected as a band of approximately 185 kDa on a PVDF membrane (Figure 1A).

To confirm that the SadA-Flag×3 protein was exposed on the surface of intact bacterial cells, a whole-cell dot blot assay was performed by incubating bacterial suspensions spotted on NCs with an anti-Flag-tag antibody. The anti-Flag-tag antibody reacted strongly with pSadA-Flag×3/StmΔ*sadA*, but not with the control group cells (Figure 1B). Since the dot blot assay did not destroy the cell membrane and the cell membrane remained intact, the result showed that the recombinant protein was successfully displayed on the surface of StmΔ*sadA* cells. At the same time, the immunofluorescence staining also confirmed that the SadA-Flag×3 protein was located on the cell surface (Figure 1C). In contrast, no fluorescence of the Flag tag was detected for pTrc99A/StmΔ*sadA*.

### 3.2. SadB Enhances the Surface Display of SadA in S. typhimurium

**Figure 2:**
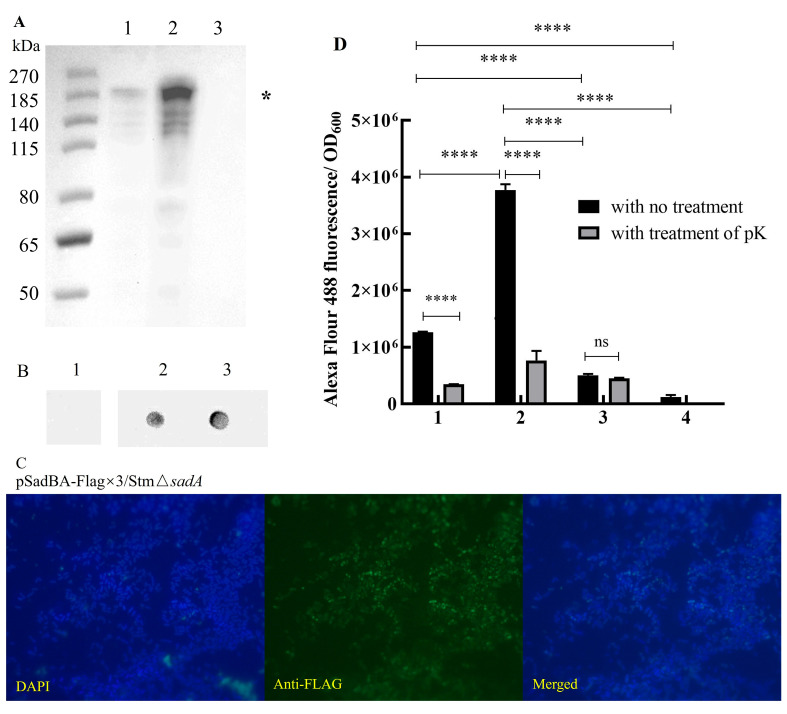
The full-length SadA fiber could display on the surface of *S. typhimurium* mutant cells with the assistance of SadB. (**A**) Western blot of whole cell lysates to analyze their expression in StmΔ*sadA*. The 1 mL induced cell suspensions (OD_600_ = 1) were washed twice by PBS and resuspended in 200 μL SDS sample buffer and boiled for 5 min. Then, 60 μL pSadA-Flag×3/StmΔ*sadA*, 30 μL pSadBA-Flag×3/StmΔ*sadA*, and 30 μL pTrc99A/StmΔ*sadA* ran on 4~12% SDS-PAGE for Western blot. The recombinant protein bands were detected by incubating the PVDF with anti-Flag-tag antibodies. The putative position of monomeric form (*) was indicated on the right side of the panel. Lane1, pSadA-Flag×3/StmΔ*sadA*; lane2, pSadBA-Flag×3/StmΔ*sadA*; lane3, pTrc99A/StmΔ*sadA*. (**B**) Dot blot of whole cells to analyze the surface display of recombinant proteins on StmΔ*sadA* with the assistance of SadB or not. Lane1, pTrc99A/StmΔ*sadA*; lane2, pSadA-Flag×3/StmΔ*sadA*; lane3, pSadBA-Flag×3/StmΔ*sadA*. (**C**) Surface display capacity as revealed by immunofluorescence staining of the Flag-tag inserted N-terminus of SadA passenger in the presence of SadB (Objective, 100×; Magnification, 1000×). (**D**) A comparison of fluorescence intensities between the whole cells treated with protease K or not. (1) pSadA-Flag×3/StmΔ*sadA*; (2) pSadBA-Flag×3/StmΔ*sadA*; (3) pFM/StmΔ*sadA*; (4) pTrc99A/StmΔ*sadA*. The data were presented as mean ± SD, and differences between groups were tested using one-way ANOVA. **** *p* < 0.0001, ns *p* > 0.05.

The *sadB* gene is located upstream of *sadA* and in an operon containing *sadA*. We created inducible overexpression constructs for the entire *sadBA* operon to improve the SadA surface display. Western blot analysis showed that the recombinant protein was successfully expressed in the presence of SadB, and the protein expression level of SadBA was significantly higher than that of SadA alone (Figure 2A).

Whole-cell dot blotting and immunofluorescence analyses showed that the recombinant proteins were located on bacterial surfaces (Figure 2B,C). Only proteins displayed on the bacterial surface can be digested by proteinase K, which cannot enter the cells. A significant decrease in the fluorescence intensity of pSadA-Flag×3/StmΔ*sadA* or pSadBA-Flag×3/StmΔ*sadA* was observed after treatment with proteinase K compared to that without treatment. Although SadA could be used for surface display, this occurred at a low level according to the fluorescence intensity (Figure 2D). The fluorescence intensity of recombinant cells in the presence of SadB was approximately three-fold higher than that in the absence of SadB (Figure 2D). These results indicated that the Flag tag was exported to the cell surface, confirming the feasibility of SadBA as a surface display vector.

### 3.3. Epitopes Can Be Displayed on the Cell Surface Using SadAs with Different Sizes as Anchoring Motifs

**Figure 3:**
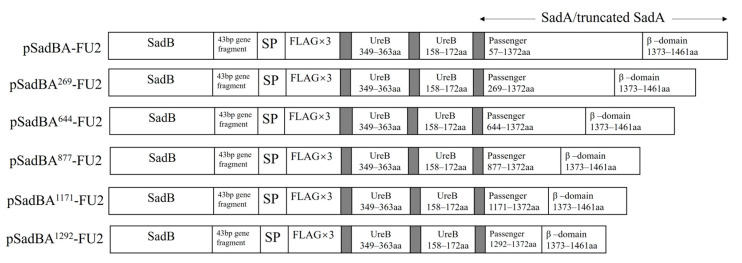
Schematic representation of genetic constructs for epitopes display using truncated and full-length SadA derivatives as an anchoring motif. SP, signal peptide of SadA; 43bp gene fragment, the gene fragment between *sadB* and *sadA* in the chromosome of *S. typhimurium*; gray, the GGGGS linker.

**Figure 4:**
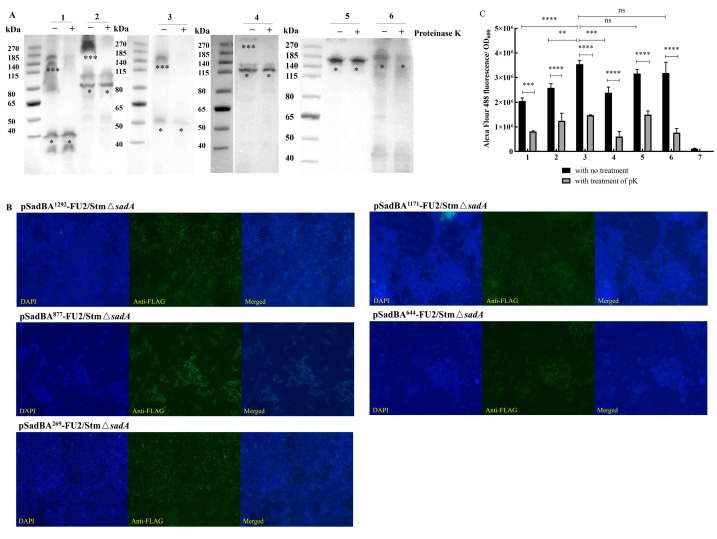
Expressing and displaying epitopes on the cell surface using full-length and truncated SadAs. (**A**) Western blot showed that fused proteins could be expressed in the StmΔ*sadA* using the antibodies against Flag tag. Then, 30 μL pSadBA^1292^-FU2/StmΔ*sadA* (lane 1), pSadBA^877^-FU2/StmΔ*sadA* (lane 2), pSadBA^1171^-FU2/StmΔ*sadA* (lane 3), pSadBA^644^-FU2/StmΔ*sadA* (lane 4), pSadBA^269^-FU2/StmΔ*sadA* (lane 5) and pSadBA-FU2/StmΔ*sadA* (lane 6) treated with proteinase K (+) or not (−) were load on 4~12% SDS-PAGE for Western blot to show the changes of bands. The putative positions of monomeric (*) and trimeric (***) complexes were indicated under the bands. (**B**) Surface display capacity as revealed by immunofluorescence staining of the Flag-tag inserted N-terminus of SadA derivatives (Objective, 100×; Magnification, 1000×). (**C**) A comparison of fluorescence intensities between the whole cells treated with protease K or not. (1) pSadBA^1292^-FU2/StmΔ*sadA*; (2) pSadBA^1171^-FU2/StmΔ*sadA*; (3) pSadBA^877^-FU2/StmΔ*sadA*; (4) pSadBA^644^-FU2/StmΔ*sadA*; (5) pSadBA^269^-FU2/StmΔ*sadA*; (6) pSadBA-FU2/StmΔ*sadA*; (7) pTrc99A/StmΔ*sadA*. The data were presented as mean ± SD, and differences between groups were tested using one-way ANOVA. ** *p* < 0.01, *** *p* < 0.001, **** *p* < 0.0001, ns *p* > 0.05.

UreB is the subunit responsible for the enzymatic activity of urease, and is considered to be an excellent candidate antigen for the development of vaccines against *H. pylori* infection. UreB158–172aa and UreB349–363aa have been identified as B-cell epitopes of UreB [23]. We fused Flag tags and the two previously mentioned epitopes of UreB with six SadA derivatives, as shown in Figure 3. These constructs were expressed in StmΔ*sadA*, and the epitopes displayed on these derivatives were examined. The expression of different SadA derivatives was detected via Western blotting using an antibody against the Flag-tag and monoclonal antibodies for UreB158–172aa (A1H10) and UreB349–363aa (A3C10), resulting in the display of monomer bands at ~40, ~50, ~80, ~115, ~160, and ~185 kDa (Figure 4A and Figure S2A,B). The bands at higher positions on the PVDF membrane could represent multimeric forms of the constructs, with the stronger reacting bands likely representing multiple conformations of dimeric and trimeric forms, which have been observed for the trimeric autotransporter YadA (*Yersinia* sp.) [24] and truncated UpaG [25]. Trimeric autotransporters have been reported to be resistant to denaturation during SDS-PAGE [26]. Expression of the trimeric form was significantly greater in the pSadBA^877^-FU2/StmΔ*sadA* strain than in the other recombinant cells (Figure 4A). The disappearance of bands corresponding to the trimeric form in pSadBA^1292^-FU2/StmΔ*sadA*, pSadBA^1171^-FU2/StmΔ*sadA*, pSadBA^877^-FU2/StmΔ*sadA*, and pSadBA^644^-FU2/StmΔ*sadA* after proteinase K treatment indicated that the recombinant proteins were displayed on the bacterial surface using SadA derivatives as the membrane anchor (Figure 4A). However, no trimer protein bands from pSadBA^269^-FU2/StmΔ*sadA* and pSadBA-FU2/StmΔ*sadA* were observed on PVDF membranes. No dot was detected in the pTrc99A/StmΔ*sadA* control group, but the specific response signal was observed with the six SadA derivatives when using the anti-Flag-tag antibody, A1H10, or A3C10, which confirmed that epitopes were successfully displayed on the surface of bacteria using the six SadA derivatives as anchoring motifs (Figure S2C).

Immunofluorescence microscopy showed a green fluorescence signal that appeared over pSadBA-FU2/StmΔ*sadA*, pSadBA^269^-FU2/StmΔ*sadA*, pSadBA^644^-FU2/StmΔ*sadA*, pSadBA^877^-FU2/StmΔ*sadA*, pSadBA^1171^-FU2/StmΔ*sadA*, and pSadBA^1292^-FU2/StmΔ*sadA* cells (Figure 4B). The display capacity was further compared by quantifying Alexa Fluor 488 fluorescence using a microplate reader. Figure 4C shows that the fluorescence intensity of pSadBA^877^-FU2/StmΔ*sadA*, pSadBA^269^-FU2/StmΔ*sadA*, and pSadBA-FU2/StmΔ*sadA* was significantly higher than that of pSadBA^1292^-FU2/StmΔ*sadA*, pSadBA^1171^-FU2/StmΔ*sadA*, and pSadBA^644^-FU2/StmΔ*sadA*, which was in line with the high rate of positivity observed in these three cell lines, according to the flow cytometric analysis (Table 1). Further, the induced recombinant *S. typhimurium* treated with proteinase K showed a significant decrease in fluorescence intensity compared to that in the absence of proteinase K (Figure 4C). These data demonstrated that epitopes were displayed on the surface of the transformed cells using truncated and full-length SadAs as the anchoring motif.

### 3.4. Truncated SadAs Can Mediate the Surface Display of Heterologous Proteins on the S. typhimurium Mutant

**Figure 5:**
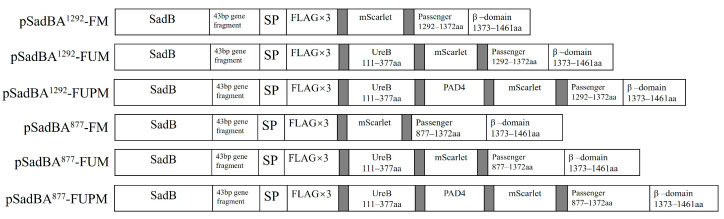
Schematic representation of genetic constructs for heterologous proteins display using truncated SadAs as an anchoring motif. SP, signal peptide of SadA; 43 bp gene fragment, the gene fragment between *sadB* and *sadA* in the chromosome of *S. typhimurium*; gray, the GGGGS linker.

The decoration of bacteria with multiple fused antigens can be used as a tool to increase the efficacy of antigen delivery. Because SadA is composed of 1462 amino acids, its full-length expression with a heterologous protein imposes a heavy burden on the cell, resulting in low expression and suboptimal surface display. Therefore, we used the truncated SadAs (SadA^1292^ and SadA^877^) to export the heterologous protein on the surface of StmΔ*sadA* cells. We chose the fluorescent protein mScarlet, the UreB fragment (UreB111–377aa, containing UreB158–172aa and UreB349–363aa), and the PAD4 fragment (139 aa), which could be used as the vaccine candidate against *B. anthracis* [27], to analyze the ability of this system to express foreign proteins. For this, we designed two truncated SadAs fused with an N-terminally Flag×3-mScarlet (~30 kDa, SadBA^877^-FM, and SadBA^1292^-FM) and Flag×3-UreBm-mScarlet (~58 kDa, SadBA^877^-FUM, and SadBA^1292^-FUM) or Flag×3-UreBm-PAD4-mScarlet (~75 kDa, SadBA^877^-FUPM, and SadBA^1292^-FUPM) (Figure 5).

**Figure 6:**
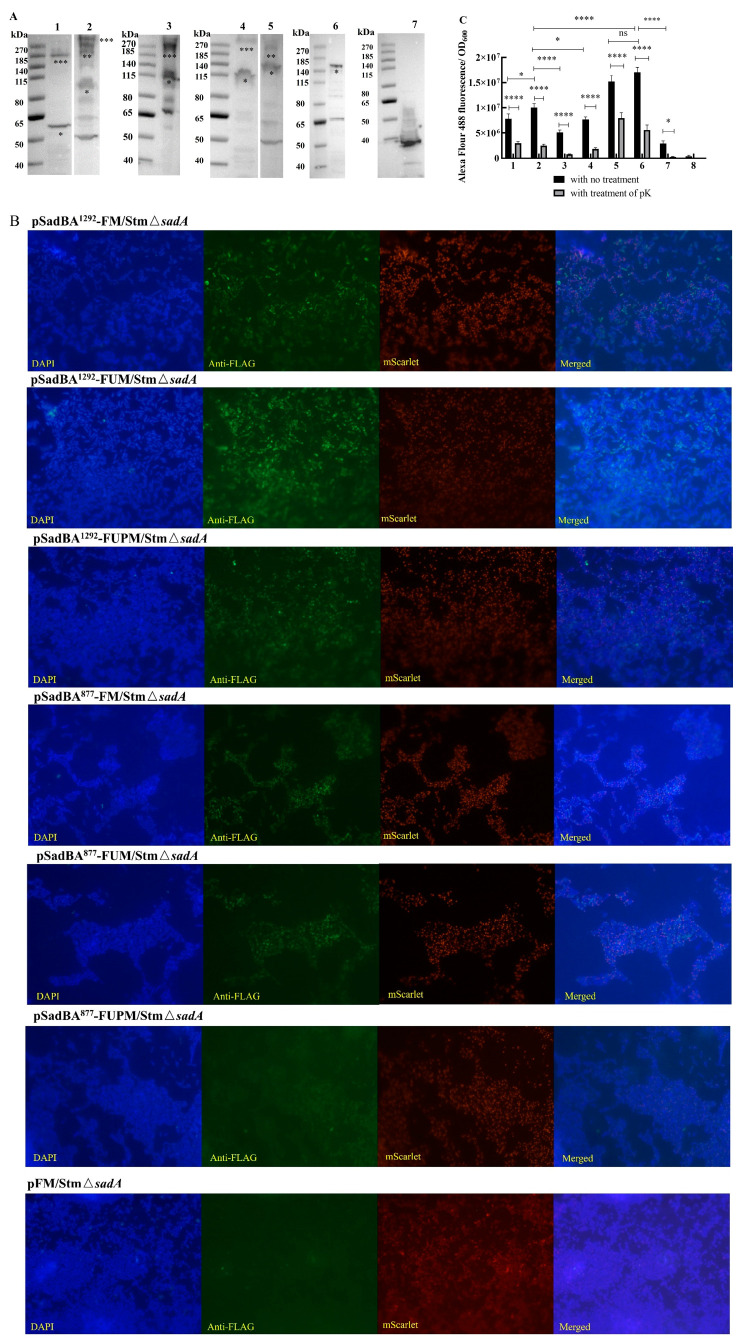
Recombinant proteins displaying on the surface of cells using truncated SadA as an anchoring motif. Western blot showed that fused proteins could be expressed in the StmΔ*sadA* using the antibodies against Flag tag (**A**). The putative positions of monomeric (*), dimeric (**), and trimeric (***) complexes were indicated under the bands. (1) pSadBA^1292^-FM/StmΔ*sadA*; (2) pSadBA^1292^-FUM/StmΔ*sadA*; (3) pSadBA^1292^-FUPM/StmΔ*sadA*; (4) pSadBA^877^-FM/StmΔ*sadA*; (5) pSadBA^877^-FUM/StmΔ*sadA*; (6) pSadBA^877^-FUPM/StmΔ*sadA*; (7) pFM/StmΔ*sadA*. (**B**) Cell surface display of Flag-tagged SadA derivatives by immunofluorescence staining using anti-Flag-tag primary antibody and Alexa Fluor 488-conjugated secondary antibody (Objective, 100×; Magnification, 1000×). (**C**) A comparison of fluorescence intensities between the whole cells treated with protease K or not. (1) pSadBA^1292^-FU2/StmΔ*sadA*; (2) pSadBA^1292^-FM/StmΔ*sadA*; (3) pSadBA^1292^-FUM/StmΔ*sadA*; (4) pSadBA^1292^-FUPM/StmΔ*sadA*; (5) pSadBA^877^-FU2/StmΔ*sadA*; (6) pSadBA^877^-FM/StmΔ*sadA*; (7) pSadBA^1292^-FUM/StmΔ*sadA*; (8) pTrc99A/StmΔ*sadA*. The data were presented as mean ± SD, and differences between groups were tested using one-way ANOVA. * *p* < 0.05, **** *p* < 0.0001, ns *p* > 0.05.

As expected, the expression of recombinant proteins resulted in a pink color when StmΔ*sadA* was induced (Figure S3A). The expression of the fusion proteins was analyzed via Western blotting using a polyclonal antibody against the Flag tag and the A1H10 monoclonal antibody (Figure 6A and Figure S3B). The monomeric bands formed by pSadBA^1292^-FM/StmΔ*sadA*, pSadBA^1292^-FUM/StmΔ*sadA*, pSadBA^1292^-FUPM/StmΔ*sadA*, pSadBA^877^-FM/StmΔ*sadA*, pSadBA^877^-FUM/StmΔ*sadA*, and pSadBA^877^-FUPM/StmΔ*sadA* were estimated to be approximately 60, 90, 115, 115, 140 and 165 kDa, respectively. And pSadBA^1292^-FM/StmΔsadA, pSadBA^1292^-FUM/StmΔ*sadA*, pSadBA^1292^-FUPM/StmΔ*sadA*, and pSadBA^877^-FM/StmΔ*sadA* resulted in trimer bands.

Whole-cell dot blot and immunofluorescence analysis indicated that the recombinant proteins were located on the surface of pSadBA^1292^-FM/StmΔ*sadA*, pSadBA^1292^-FUM/StmΔ*sadA*, pSadBA^1292^-FUPM/StmΔ*sadA*, pSadBA^877^-FM/StmΔ*sadA*, and pSadBA^877^-FUM/StmΔ*sadA* (Figures S3C and 6B). However, a small amount of green fluorescence was observed on pSadBA^877^-FUPM/StmΔ*sadA*, indicating that FUPM display on the cell surface was marginal when using SadBA^877^ as the anchoring motif. No fluorescence was detected for pFM/StmΔ*sadA*, which is a control strain, expressing FM with no membrane anchor.

The fluorescent intensity and rates of positivity of SadBA^877^-FUM/StmΔ*sadA* were significantly lower than those of pSadBA^877^-FM/StmΔ*sadA* (Figure 6C, Table 1). Meanwhile, the fluorescence intensity of pSadBA^1292^-FM/StmΔ*sadA* was higher than that of pSadBA^1292^-FUM/StmΔ*sadA* and pSadBA^1292^-FUPM/StmΔ*sadA*. These results indicated that the SadA display was decreased as the molecular weight of the foreign protein was increased.

The fluorescence intensity and rate of positivity of pSadA^877^-FM/StmΔ*sadA* were higher than those of pSadBA^1292^-FM/StmΔ*sadA* (Figure 6C, Table 1), which was consistent with the improved ability of SadBA^877^ to facilitate epitope surface display compared to that of SadBA^1292^.

When the recombinant cells were treated with proteinase K, the fluorescence of pSadBA^1292^-FM/StmΔ*sadA*, pSadBA^1292^-FUM/StmΔ*sadA*, pSadBA^1292^-FUPM/StmΔ*sadA*, pSadBA^877^-FM/StmΔ*sadA*, and pSadBA^877^-FUM/StmΔ*sadA* was decreased significantly (Figure 6C). These data indicated that the heterologous proteins FM, FUM, and FUPM could be displayed on the surfaces of transformant cells using SadBA^1292^ as an anchoring motif and that SadA^877^ could help to expose FM and FUM on the surfaces of cells.

### 3.5. Immune Responses Elicited by pSadBA^1292^-FUM/StmΔygeAΔmurI and pSadBA^877^-FUM/StmΔygeAΔmurI in Mice Model

**Figure 7:**
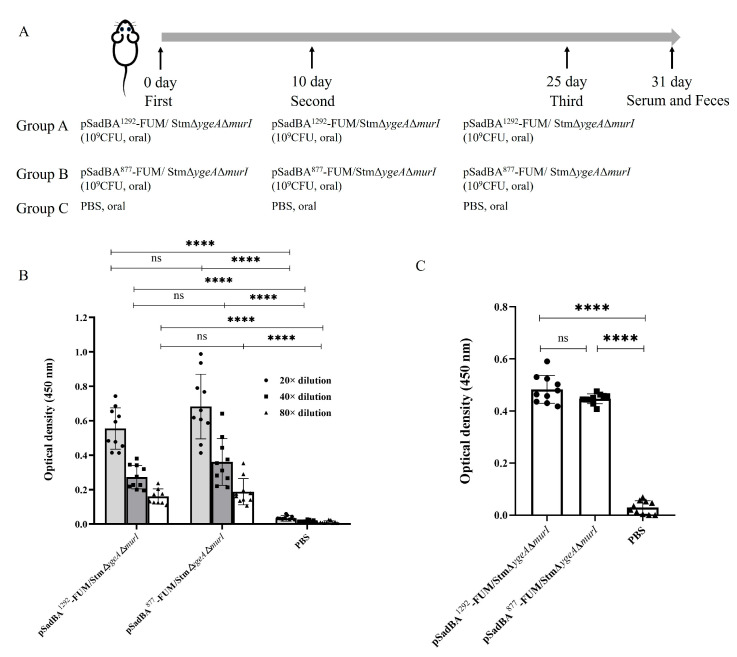
Immunization schedule and immune responses induced by immunization. (**A**) Group A and B mice received oral administration of 10^9^ CFU pSadBA^1292^-FUM/StmΔ*ygeA*Δ*murI* and pSadBA^877^-FUM/StmΔ*ygeA*Δ*murI* on days 0, 10, 25, respectively; Group C mice received oral administration of PBS. On the sixth day after the final immunization, serum and feces of the mice were collected and the UreB-specific IgG (**B**) and sIgA (**C**) levels were measured by ELISA. Differences between groups were tested using one-way ANOVA. **** *p* < 0.0001, ns *p* > 0.05.

We transferred pSadBA^1292^-FUM and pSadBA^877^-FUM into competence cells of StmΔ*ygeA*Δ*murI*, which had been confirmed by PCR and sequencing (Figure S1B). The heterologous proteins were expressed and the surface displayed successfully in StmΔ*ygeA*Δ*murI* (Figures S4 and S5). In order to determine whether the recombinant protein was able to elicit antibodies, BALB/c mice were immunized orally with pSadBA^1292^-FUM/StmΔ*ygeA*Δ*murI* and pSadBA^877^-FUM/StmΔ*ygeA*Δ*murI* (Figure 7A). Antigen-specific IgGs of groups A and B were significantly higher than those of group C in the same dilution. Meanwhile, differences in serum IgG levels for groups A and B were not significant in the same dilution (Figure 7B). Compared with the control group C, groups A and B both produced the antigen-specific mucosal sIgA, but there was no difference between groups A and B (Figure 7C).

## 4. Discussion

SadA was the first reported TAA exposed on the surface of *Salmonella* spp. cells and it is highly conserved among *S. enterica* strains [18]. SadA is a positional ortholog of UpaG and EhaG in *E. coli*, but with different functions. Specifically, it promotes biofilm formation and host cell adherence but does not bind extracellular matrix molecules or mediate serum resistance. Although the exact mechanism of TAA secretion remains unclear, many important proteins involved in TAA biogenesis have been found, such as BAM [28] and chaperones [29]. During the biogenesis of SadA, a trimeric protein, SadB, for which the encoding gene is located upstream of sadA, facilitates the export of SadA to the cell surface. pSadBA-Flag×3/StmΔ*sadA* had an approximately three-fold higher mean fluorescence than pSadA-Flag×3/StmΔ*sadA* in this study (Figure 2D), which is in accord with a previous report [15]. Western blot results showed that SadA monomer expression in pSadBA-Flag×3/StmΔ*sadA* was significantly higher than that in pSadA-Flag×3/StmΔ*sadA* in this study, suggesting that SadB might enhance the surface display of SadA by increasing its expression or preventing its degradation when it is not yet at the cell surface, in the periplasm. During TAA biogenesis, the N-terminal signal sequence mediates translocation of the subunit into the periplasm in a Sec-dependent manner. The Sec system also transports several proteins, such as lipoproteins, periplasmic proteins, and outer membrane proteins (OMPs), into the periplasm [30]. When heterologous OMPs are over-expressed, the Sec machinery is prone to saturation. To solve this problem, the most abundant OMPs can be knocked out to alleviate the burden on the Sec system and provide a larger membrane area for foreign proteins. Meuskens found that the expression of the membrane anchor domain of the trimeric autotransporter YadA (YadAM) is higher in *ompA-ompC-ompF-lamB* quadruple mutant BL21 Gold (DE3) cells than in BL21 (DE3) cells. This provides ideas for further improving the SadA display [31].

Nakatani et al. found that in bacteria, a His-tag displayed by full-length AtaA could bind to Ni-Sepharose beads more rapidly than other truncated AtaAs, confirming that a certain distance from the bacterial surface could help to overcome steric hindrance and maintain the function of foreign proteins [14]. Possibly for the same reason, pSadBA^1292^-FU2/StmΔ*sadA*, pSadBA^1171^-FU2/StmΔ*sadA*, and pSadBA^644^-FU2/StmΔ*sadA* exhibited a significantly lower fluorescence intensity than pSadBA-FU2/StmΔ*sadA* and pSadBA^269^-FU2/StmΔ*sadA* in this study (Figure 4C). This indicates that the exogenous peptides displayed by full-length SadA are more easily recognized by antibodies. However, although SadA^877^ is shorter, the expression of its trimer was higher (Figure 4A). Accordingly, the fluorescence intensity of pSadBA^877^-FU2/StmΔ*sadA* was significantly enhanced, which was similar to that with pSadBA-FU2/StmΔsadA and pSadBA^269^-FU2/StmΔ*sadA* (Figure 4C).

An autotransporter-based display system can display a protein of interest (POI) on the surface of gram-negative bacteria through the exchange of all or part of the native passenger domain with the POI. However, one limiting factor for the successful translocation of autotransporters is the final dimensions of the fused protein [32]. For example, the maximum size limit of heterologous proteins using AIDA-I of *E. coli*, MisL of *S. typhimurium*, IcsA of *Shigella* spp., and Hbp of *E. coli* is 130 kDa [33], 58 kDa [34], 57 kDa [35], and 50 kDa [36], respectively. In this study, SadBA^1292^ could be used to display FUPM (~75 kDa) on the surface of *S. typhimurium*. However, with SadBA^877^, the display of FUPM (~75 kDa), which is larger than FUM (~58 kDa), on the bacterial surface, failed (Figure 6B). Moreover, the fluorescence intensity of pSadBA^877^-FUM/StmΔ*sadA* and pSadBA^1292^-FUM/StmΔ*sadA* was significantly lower than that of pSadBA^877^-FM/StmΔ*sadA* and pSadBA^1292^-FM/StmΔ*sadA*, respectively. As the molecular weight of the foreign protein increases, it becomes more difficult to display the recombinant protein on the cell surface. The complex structure of foreign proteins is another limiting factor for the successful exposure of autotransporters [37]. When autotransporter Hbp was used to display calmodulin or a nanobody, secretion was hindered because the recombinant protein could not pass through the β-barrel due to the form of a stable fold in the presence of calcium ions or the form of two disulfide bonds [38]. BamA and BamD are required for the secretion and function of SadA [39]. In contrast, SadA can be displayed on the surfaces of mutant cells in which *bamB*, *bamC*, or *bamE* are deleted, suggesting that these three lipoproteins are not essential for SadA secretion. Trang et al. found that overproduction of the BAM complex can increase the surface display of Hbp fusions and the trimeric autotransporter UpaG [15]. This provides a solution for the secretion of heterologous proteins with complex structures using SadA as a surface display tool.

Autotransporters (type Va secretion systems) have been widely used to display foreign proteins on cell surfaces, especially for vaccine development. AIDA-I, MisL, ShdA, and Hbp have been successfully used to display different antigens on the surfaces of *Salmonella* spp. as vaccine candidates. Moreover, auto-displayed vaccines can effectively increase the immunogenicity of antigens [40]. TAA belongs to the T5cSS family and exhibits auto-display characteristics similar to those of autotransporters. In contrast to autotransporters, TAAs can display stable trimeric polymers on gram-negative bacterial surfaces owing to the structure of T5cSS. Trimeric proteins exhibited a stronger immunogenic response than their monomer forms [41].

Genetically attenuated *Salmonella* spp. can be engineered by deleting important virulence genes and the key enzyme genes of metabolic pathways to deliver recombinant heterologous antigens to elicit the host immune system [42]. D-Glu, the major component of peptidoglycan, is synthesized by MurI and YgeA in *Salmonella* spp. A *murI*-deleted *S. typhimurium* displayed a lower level of virulence than the wild-type strain and preserved pathogen-associated molecular patterns [43]. Cabral et al. found that the D-Glu auxotrophic *Pseudomonas aeruginosa* could evoke an immune response with intranasal administration and protect the mice infected with two cytotoxic *P. aeruginosa* strains [44]. In this work, a mutant strain StmΔ*ygeA*Δ*murI* was constructed and employed to confirm the immunogenicity of heterologous antigen displayed by SadA. Orally administrated pSadBA^1292^-FUM/StmΔ*ygeA*Δ*murI* or pSadBA^877^-FUM/StmΔ*ygeA*Δ*murI* can elicit a mucosal and humoral immune response. Therefore, the surface-displayed antigen using truncated SadA as an anchoring motif kept its antigenicity and evoked host immunity reactivity. SadA could be exploited to construct a novel antigen surface expression system for attenuated bacterial vaccine development.

## 5. Conclusions

In summary, the SadA display was tested in terms of its length and the heterologous protein load. Both full-length and truncated SadAs could display exogenous proteins. In vivo test, the heterologous antigen surface displayed on StmΔ*ygeA*Δ*murI* using truncated SadA as an anchoring motif could elicit a significant mucosal and humoral immune response. These findings suggest that SadA anchoring proteins are suitable for developing an attenuated bacterial vaccine.

## Figures and Tables

**Table 1:** Flow cytometric analysis of different SadA derivatives.

Sample	Positive Rate (%)			Average Value
	1	2	3	
pTrc99A/StmΔ*sadA*	0.02%	0.00%	0.00%	0.0067%
pSadBA^1292^-FU2/StmΔ*sadA*	71.55%	77.22%	73.85%	74.21%
pSadBA^1171^-FU2/StmΔ*sadA*	41.17%	32.41%	41.13%	38.24%
pSadBA^877^-FU2/StmΔ*sadA*	78.51%	83.99%	82.55%	81.68%
pSadBA^644^-FU2/StmΔ*sadA*	50.39%	52.05%	55.16%	52.53%
pSadBA^269^-FU2/StmΔ*sadA*	85.70%	86.50%	87.90%	86.70%
pSadBA-FU2/StmΔ*sadA*	85.00%	87.37%	89.08%	87.15%
pSadBA^1292^-FM/StmΔ*sadA*	85.77%	83.46%	84.43%	84.55%
pSadBA^1292^-FUM/StmΔ*sadA*	76.30%	75.26%	74.08%	75.21%
pSadBA^1292^-FUPM/StmΔ*sadA*	82.55%	82.75%	85.26%	83.52%
pSadBA^877^-FM/StmΔ*sadA*	92.47%	93.36%	93.43%	93.09%
pSadBA^877^-FUM/StmΔ*sadA*	32.04%	33.12%	41.23%	35.46%

## Data Availability

Data are contained within the article and supplementary material. Further inquiries can be directed to the corresponding authors.

## Supplementary Materials

The following supporting information can be downloaded at: https://www.mdpi.com/article/10.3390/vaccines12040399/s1, Table S1: bacterial strains and plasmids; Table S2: oligonucleotides used in this study; Table S3: the gene sequences of commercial synthesis and amino acid sequences of functional proteins; Figure S1: the sadA-deleted S. typhimurium and ygeA-murI-double-deleted S. typhimurium were confirmed by PCR; Figure S2: Western blot and dot blot of the displaying epitopes on the cell surface using full-length and truncated SadAs; Figure S3: confirmation of recombinant proteins displaying on the surface of cells using truncated SadAs as an anchoring motif; Figure S4: Western blot of heterologous proteins expressed in StmΔygeAΔmurI using the antibodies against Flag tag (A) and UreB158-172aa (B); Figure S5: cell surface display of Flag-tagged SadA derivatives on StmΔygeAΔmurI by immunofluorescence staining using anti-Flag-tag primary antibody and Alexa Fluor 488 conjugated secondary antibody.
