# Supplementary material for: The Trimeric Autotransporter Adhesin SadA from Salmonella spp. as a Novel Bacterial Surface Display System
Source: Vaccines (Basel). 2024 Apr 9;12(4):399. doi: 10.3390/vaccines12040399 (PMC11054257; doi:10.3390/vaccines12040399)
Supplement: Supplementary file 1 [file vaccines-12-00399-s001.zip › supplementary materials.pdf]

Table S1. Bacterial strains and plasmids.

Strain or plasmid	Genotype or description	Reference or source
Stains		
DH5α	Cloning strain	CWBIO, China
S. typhimurium 1.1174	Wild-type *S. typhimurium*	This laboratory
Stm*ΔSadA*	*S. typhimurium* 1.1174 with *sadA* gene deletion	This work
B. anthracis A16R	pXO1+pXO2–, China vaccine strain, the strain for cloning the gene of PAD4.	This laboratory
H. pylori SS1	Strain for cloning the gene of UreB.	This laboratory
Stm*ΔygeAΔmurI*	*S. typhimurium* 1.1174 with *ygeA* and *murI* genes double-deletion	This work
Plasmids		
pUC57	*amp*, P_{lac}, used for synthesizing gene fragments commercially	Generalbiol, China
pTrc99A	*amp*, P_{trc}, used for expressing the heterologous proteins with different SadA derivatives	Lab collection
pSadA-Flag×3	pTrc99A derivative containing *sadB* and *sadA* genes fused with the gene 3×Flag-tag	This work
pSadBA-Flag×3	*pTrc99A* derivative containing *sadB* and *sadA* genes fused with the gene of 3×Flag-tag	This work
pSadBA-FU2	*pTrc99A* derivative containing *sadB* and *sadA* genes fused with genes of 3×Flag-tag, UreB158-172 and UreB349–363 from *H. pylori* SS1	This work
pSadBA^{1292}-FU2	*pTrc99A* derivative containing *sadB* and *sadA*$^{1292\text{-}1462aa}$ genes fused with genes of 3×Flag-tag, UreB158-172 and UreB349–363 from *H. pylori* SS1	This work
pSadBA^{1171}-FU2	*pTrc99A* derivative containing *sadB* and *sadA*$^{1171\text{-}1462aa}$ genes fused with genes of 3×Flag-tag, UreB158-172 and UreB349–363 from *H. pylori* SS1	This work
pSadBA^{877}-FU2	*pTrc99A* derivative containing *sadB* and *sadA*$^{877\text{-}1462aa}$ genes fused with genes of 3×Flag-tag, UreB158-172 and UreB349–363 from *H. pylori* SS1	This work
pSadBA^{644}-FU2	*pTrc99A* derivative containing *sadB* and *sadA*$^{644\text{-}1462aa}$ genes fused with genes of 3×Flag-tag, UreB158-172 and UreB349–363 from *H. pylori* SS1	This work
pSadBA^{269}-FU2	*pTrc99A* derivative containing *sadB* and *sadA*$^{269\text{-}1462aa}$ genes fused with genes of 3×Flag-tag, UreB158-172 and UreB349–363 from *H. pylori* SS1	This work
pSadBA-FU2	*pTrc99A* derivative containing *sadB* and *sadA* genes fused with genes of 3×Flag-tag, UreB158-172 and UreB349–363 from *H. pylori* SS1	This work
pSadBA^{1292}-FM	*pTrc99A* derivative containing *sadB* and *sadA*$^{1292\text{-}1462aa}$	This work

	genes fused with genes of 3×Flag-tag and mScarlet	
pSadBA^{1292}-FUM	*pTrc99A* derivative containing *sadB* and *sadA*$^{1292\text{-}1462aa}$ genes fused with genes of 3×Flag-tag, UreBm (111-376 aa) and mScarlet	This work
pSadBA^{1292}-FUPM	*pTrc99A* derivative containing *sadB* and *sadA*$^{1292\text{-}1462aa}$ genes fused with genes of 3×Flag-tag, UreB111-376 from *H. pylori* SS1, PAD4 from *B. anthracis* and mScarlet	This work
pSadBA^{877}-FM	*pTrc99A* derivative containing *sadB* and *sadA*$^{877\text{-}1462aa}$ genes fused with genes of 3×Flag-tag and mScarlet	This work
pSadBA^{877}-FUM	*pTrc99A* derivative containing *sadB* and *sadA*$^{877\text{-}1462aa}$ genes fused with genes of 3×Flag-tag, UreBm (111-376 aa) and mScarlet	This work
pSadBA^{877}-FUPM	*pTrc99A* derivative containing *sadB* and *sadA*$^{877\text{-}1462aa}$ genes fused with genes of 3×Flag-tag, UreB111-376 from *H. pylori* SS1, PAD4 from *B. anthracis* and mScarlet	This work
pFM	*pTrc99A* derivative containing genes of 3×Flag-tag and mScarlet without an anchoring motif	This work
pJOE-mScarlet	Deriving from pJOE8999, from 1 to 2844 base pairs, carrying mScarlet coded sequence without promoter	Lab collection
pCas	*repA101*(Ts), *kan*, P_{cas}-*cas9*, P_{araB}-Red, P_{trc}-sgRNA-*pMB1*, used for *sadA* gene deletion	Jiang etal.
pTargetF-*sadA*	*pMB1*, *aadA:* spectinomycin resistance gene, sgRNA-*sadA*, used for *sadA* gene deletion	Jiang etal.
pTargetFA-*ygeA*	*pMB1*, *bla:* ampicillin resistance gene, sgRNA-*ygeA*, used for *ygeA* gene deletion	Jiang etal.
pTargetFA-*murI*	*pMB1*, *bla:* ampicillin resistance gene, sgRNA-*murI*, used for *murI* gene deletion	Jiang etal.

Yu Jiang, Biao Chen, Chunlan Duan, Bingbing Sun, Junjie Yang, Sheng Yang. Multigene Editing in the Escherichia coli Genome via the CRISPR-Cas9 System. Appl Environ Microbiol, 2015, 81(7): 2506-14.

Table S2. Oligonucleotides used in this study.

Name	Sequence	Target
sadA-F	CGGGATCCATGAATAGAATATTTAAAGTCCTCTGGAA	*sadA*$^{1–168}$-*flag*×3-*sadA*$^{169–990}$; *sadA*$^{1–168}$-*flag*×3-*a3c10*-*a1h10* -*sadA*$^{169–990}$
sadA$^{1-990}$-R	CTAGCTAGCGCTAAAGGCGCTGATGCTTT	*sadA*$^{1–168}$-*flag*×3-*sadA*$^{169–990}$; *sadA*$^{1–168}$-*flag×3-a3c10-a1h10* -*sadA*$^{169–990}$
sadA$^{991-4386}$-F	CTAGCTAGCCACACGGGAAACGCCAGCAAAAT	*sadA*$^{991–4386}$
sadA-R	CCCAAGCTTTTACCACTGGAAGCCCGCG	*sadA*$^{991–4386}$
sadBA-F	CCGGAATTCATGCACAAAAATGGAAAATTT	*sadB*-*sadA*$^{1–168}$-*flag*×3-*sadA*$^{169–4386}$
sadBA-R	ATCCTTGTAATCTGCGTTGGCCAGTGCATCCGCAGAAACC	*sadB*-*sadA*$^{1–168}$-*flag*×3-*sadA*$^{169–4386}$
sadBA-flag×3-F	ACTGGCCAACGCAGATTACAAGGATGACGACGATAAGG	*sadB*-*sadA*$^{1–168}$-*flag*×3-*sadA*$^{169–4386}$
sadBA-flag×3-R	*sadA*-R	*sadB*-*sadA*$^{1–168}$-*flag*×3-*sadA*$^{169–4386}$
sadBA^{1292}-*FU*2-1-F	*sadB*-F	*sadBA*^{1292}-*FU*2
sadBA^{1292}-*FU*2-1-R	GTTCACCCCTGCACTTCCTCCTCCTCCGATAGTAG	*sadBA*^{1292}-*FU*2
sadBA^{1292}-*FU*2-2-F	GGAGGAGGAAGTGCAGGGGTGAACAACACTGA	*sadBA*^{1292}-*FU*2
sadBA^{1292}-*FU*2-2-R	*sadA*-R	*sadBA*^{1292}-*FU*2
sadBA^{1171}-*FU*2-1-F	*sadB*-F	*sadBA*^{1171}-*FU*2
sadBA^{1171}-*FU*2-1-R	TGCCGAACCCGCACTTCCTCCTCCTCCGATAGTAG	*sadBA*^{1171}-*FU*2
sadBA^{1171}-*FU*2-2-F	GGAGGAGGAAGTGCGGGTTCGGCAGATACCGAT	*sadBA*^{1171}-*FU*2
sadBA^{1171}-*FU*2-2-R	*sadA*-R	*sadBA*^{1171}-*FU*2
sadBA^{877}-*FU*2-1-F	*sadB*-F	*sadBA*^{877}-*FU*2
sadBA^{877}-*FU*2-1-R	GATTTCACCTGCACTTCCTCCTCCTCCGATAGTAG	*sadBA*^{877}-*FU*2
sadBA^{877}-*FU*2-2-F	GGAGGAGGAAGTGCAAGGTGAAATCGCCAGTGA	*sadBA*^{877}-*FU*2
sadBA^{877}-*FU2*-2-R	*sadA*-R	*sadBA*^{877}-*FU*2
sadA^{644}-*FU*2-1-F	*sadB*-F	*sadBA*^{644}-*FU*2
sadBA^{644}-*FU*2-1-R	CAGGGTGCCCGCACTTCCTCCTCCTCCGATAGTAG	*sadBA*^{644}-*FU*2
sadBA^{644}-*FU*2-2-F	GGAGGAGGAAGTGCGGGCACCCTGGCCGCGGACA	*sadBA*^{644}-*FU*2
sadBA^{644}-*FU*2-2-R	*sadA*-R	*sadBA*^{644}-*FU*2
sadBA^{269}-*FU*2-1-F	*sadB*-F	*sadBA*^{269}-*FU*2
sadBA^{269}-*FU*2-1-R	CTATTAACGGAAGCACTTCCTCCTCCTCCGATAGTAG	*sadBA*^{269}-*FU*2
sadBA^{269}-*FU*2-2-F	GGAGGAGGAAGTGCTTCCGTTAATAGTGATGGTA	*sadBA*^{269}-*FU*2
sadBA^{269}-*FU*2-2-R	*sadA*-R	*sadBA*^{269}-*FU*2
sadBA-*FU*2-1-F	*sadB*-F	*sadBA*-*FU*2
sadBA-*FU*2-1-R	TGTATCGTTTCCACTTCCTCCTCCTCCGATAGTAGTCGCGTT	*sadBA*-*FU*2
sadBA-*FU*2-2-F	ATCGGAGGAGGAGGAAGTGGAAACGATACAGGCGAC	*sadBA*-*FU*2

	GGC	
*sadBA-FU*2-2-R	*sadA*-R	*sadBA-FU*2
sadBA^{1292}-*FM*-1-F	*sadB*-F	*sadBA*^{1292}-*FM*
sadBA^{1292}-*FM*-1-R	ACCTTTAGATACACTTCCTCCTCCTCCTTTA	*sadBA*^{1292}-*FM*
sadBA^{1292}-*FM*-2-F	AGGAGGAGGAAGTGTATCTAAAGGTGAAGCAGTAA	*sadBA*^{1292}-*FM*
sadBA^{1292}-*FM*-2-R	ACTTCCTCCTCCTCCTTTGTATAATTCATCCAT	*sadBA*^{1292}-*FM*
sadBA^{1292}-*FM*-3-F	GATGAATTATACAAAGGAGGAGGAGGAAGTGCA	*sadBA*^{1292}-*FM*
sadBA^{1292}-*FM*-3-R	*sadA*-R	*sadBA*^{1292}-*FM*
sadBA^{1292}-*FUM*-1-F	*sadB*-F	*sadBA*^{1292}-*FUM*
sadBA^{1292}-*FUM*-1-R	ACGCTAAGATTACTTCCTCCTCCTCCTTT	*sadBA*^{1292}-*FUM*
sadBA^{1292}-*FUM*-2-F	GAGGAGGAAGTAATCTTAGCGTGGGTCCTGCTA	*sadBA*^{1292}-*FUM*
sadBA^{1292}-*FUM*-2-R	CTTTAGATACACTTCCTCCTCCTCCCCAAGTTCTAGTGATAA	*sadBA*^{1292}-*FUM*
sadBA^{1292}-*FUM*-3-F	TAGAACTTGGGGAGGAGGAGGAAGTGTATCTAAAGGTGAAGCA	*sadBA*^{1292}-*FUM*
sadBA^{1292}-*FUM*-3-R	*sadA*-R	*sadBA*^{1292}-*FUM*
sadBA^{1292}-*FUPM*-1-F	*sadB*-F	*sadBA*^{1292}-*FUPM*
sadBA^{1292}-*FUPM*-1-R	TCATAATGAAA ACTTCCTCCTCCTCCTTTATCGTCATCATC	*sadBA*^{1292}-*FUPM*
sadBA^{1292}-*FUPM*-2-F	*sadBA*^{1292}-*FUM*-2-F	*sadBA*^{1292}-*FUPM*
sadBA^{1292}-*FUPM*-2-R	TGAAAACTTCCTCCTCCTCCCCAAGTTCTAGTGATAA	*sadBA*^{1292}-*FUPM*
sadBA^{1292}-*FUPM*-3-F	TTGGGGAGGAGGAGGAAGTTTTCATTATGATAGAAATAACA	*sadBA*^{1292}-*FUPM*
sadBA^{1292}-*FUPM*-3-R	GATACACTTCCTCCTCCTCCTCCTATCTCATAGCCTT	*sadBA*^{1292}-*FUPM*
sadBA^{1292}-*FUPM*-4-F	TAGGAGGAGGAGGAGGAAGTGTATCTAAAGGTGAAGCA	*sadBA*^{1292}-*FUPM*
sadBA^{1292}-*FUPM*-4-R	*sadA*-R	*sadBA*^{1292}-*FUPM*
sadBA^{877}-*FM*-1-F	*sadB*-F	*sadBA*^{877}-*FM*
sadBA^{877}-*FM*-1-R	TTTCACCTGCACTTCCTCCTCCTCC TTTGTATAATTCATCC	*sadBA*^{877}-*FM*
sadBA^{877}-*FM*-2-F	GAGGAGGAAGTGCAGGTGAAATCGCCAGT	*sadBA*^{877}-*FM*
sadBA^{877}-*FM*-2-R	*sadA*-R	*sadBA*^{877}-*FM*
sadBA^{877}-*FUM*-1-F	*sadB*-F	*sadBA*^{877}-*FUM*
sadBA^{877}-*FUM*-1-R	*sadBA*^{1292}-*FUM*-1-R	*sadBA*^{877}-*FUM*
sadBA^{877}-*FUM*-2-F	*sadBA*^{1292}-*FUM*-2-F	*sadBA*^{877}-*FUM*
sadBA^{877}-*FUM*-2-R	*sadBA*^{1292}-*FUM*-2-R	*sadBA*^{877}-*FUM*
sadBA^{877}-*FUM*-3-F	*sadBA*^{1292}-*FUM*-3-F	*sadBA*^{877}-*FUM*
sadBA^{877}-*FUM*-3-R	*sadA*-R	*sadBA*^{877}-*FUM*
sadBA^{877}-*FUPM*-1-F	*sadB*-F	*sadBA*^{877}-*FUM*
sadBA^{877}-*FUPM*-1-R	*sadBA*^{877}-*FM*-1-R	*sadBA*^{877}-*FUM*
sadBA^{877}-*FUPM*-2-F	*sadBA*^{877}-*FM*-2-F	*sadBA*^{877}-*FUM*
sadBA^{877}-*FUPM*-2-R	*sadA*-R	*sadBA*^{877}-*FUM*
Up-*sadA*-F	AACACAACCAAAAAGGTCACCGAAGTCG	Upstream homologous arm of *sadA*

Up-*sadA*-R	GTTGTTACTTCAGAATGAGTAATTTCCT	Upstream homologous arm of *sadA*
Down-*sadA*-F	ATTACTCATTCTGAAGTAACAACACTCCC	Downstream homologous arm of *sadA*
Down-*sadA*-R	CGCGTGATCGACCGGCATTTTTATAGAACA	Downstream homologous arm of *sadA*
△*sadA*-test-F	CTTATATCCCGCAGAATATG	Test for *sadA* gene deletion
△*sadA*-test-R	GAATCTTTGCTCATCTCGTTG	Test for *sadA* gene deletion
ygeA-F	GCGCCACGACTTAGGGCTGACGGAGA	Test for homologous arm of *ygeA*
ygeA-R	CATTGGTGTTGGGCTACTGCCTGATGCA	Test for homologous arm of *ygeA*
△*ygeA*-test-F	AATCCTTTTTGCCGCCGTTACTACCGCCTTTT	Test for *ygeA* gene deletion
△*ygeA*-test-R	TCAAACTCAAGCAGGGAGGGTGGGCATTAT	Test for *ygeA* gene deletion
Up-*murI*-F	GTCAGCTGGCGTATTTCAGGTTATCGTAA	Upstream homologous arm of *murI*
Up-*murI*-R	CCCAAAACGCCATCAGAAGGTGTAGCTGCCAGACA	Upstream homologous arm of *murI*
Down-*murI*-F	TACACCTTCTGATGGCGTTTTGGGTAAATACCAGGC	Downstream homologous arm of *murI*
Down-*murI*-R	GCGGTATTAGCCACCGTTTCCAGTAGTTA	Downstream homologous arm of *murI*
△*murI*-test-F	TTTATTgCCTCCTACggAACCTCCTACAAA	Test for *murI* gene deletion
△*murI*-test-R	TAATTTCTCAAgTgCCTggTATTTACCCAAAACgC	Test for *murI* gene deletion

Table S3. The commercial synthetic gene sequences and amino acid sequences of functional proteins parts used in this study.

sadA$^{1–168}$-*flag*×3-*sadA*$^{169–330}$<br>(green, *flag*×3)	atgaatagaatatttaaagtcctctggaatgccgctacgggaacatttgttgtcaccagcgaaacc<br>gcaaaaagccgcggcaaaaaaaacggccgcagaaagctggcagtttccgcactcatcggtctt<br>agcagcattatggtttctgcggatgcactggccaacgcagattacaaggatgacgacgataagg<br>actataaggacgatgatgacaaggactacaaagatgatgacgataaaggaaacgatacaggcg<br>acggcgttactccaacgggtacccagactggaggaaaagggtggattgcaattggtaccgatgc<br>cacagccaatacttacaccaacgttgatggcgcaagcgccgcaatgggttataaagcctccgcg<br>atggggaaatggagtaccgccattggttcctacagccagtccaccggcgactcttcgttagcgctt<br>ggcgtaaaatcggtttcagccggtgaccgggccattgcaatgggcgcctcatcctcagccagtg<br>gaagttattcaatggcaatgggcgtgtatgccaattcgagcggcgcaaaatcggttgcgttaggtt<br>ataaatctgtcgcgagcggagcaacttcctctgcattaggttatcaagctactgcgagcggcgac<br>gacagcgctgcttttggtaatggcgcaaaagcgataggcaccaactcagttgcccttggctcggg<br>ctctgtcgcccaggaagacaattccgtcgccgtgggtaacagcaccactcagcggcagataacc<br>tacgttgctaaaggcgacatcaattccaccagtaccgatgccgttacaggtgcgcaaatttattcttt<br>aagtcaatccgtcgccgaccgactcggcggaggggcttccgttaatagtgatggtacagtgaat<br>gcgcccctctacgaggtaggcacaggcatctacaataacgtaggtagtgcattaagcgcacttaa<br>cacgtctatcactaacacagaggcctctgtcgcaggattagccgaagacgcgctgttgtgggatg<br>aaagcatcagcgcctttagcgctagc
sadA$^{1–168}$-*flag*×3-*a3c10*-*a1h10*-*sadA*$^{169–990}$<br>(green, *flag*×3; yellow, the gene of UreB349–363; blue, the gene of UreB158–172; red, the gene of linker (GGGGS))	atgaatagaatatttaaagtcctctggaatgccgctacgggaacatttgttgtcaccagcgaaacc<br>gcaaaaagccgcggcaaaaaaaacggccgcagaaagctggcagtttccgcactcatcggtctt<br>agcagcattatggtttctgcggatgcactggccaacgcagattacaaggatgacgacgataagg<br>actataaggacgatgatgacaaggactacaaagatgatgacgataaaGGAGGAGGAG<br>GAAGTactttgcatgacatggggattttctcaatcactagttctgactctGGAGGAGGA<br>GGAAGTggtggcggaactggtcctgctgatggcactaacgcgactactatcGGAGGA<br>GGAGGAAGTggaaacgatacaggcgacggcgttactccaacgggtacccagactgga<br>ggaaaagggtggattgcaattggtaccgatgccacagccaatacttacaccaacgttgatggcg<br>caagcgccgcaatgggttataaagcctccgcgatggggaaatggagtaccgccattggttccta<br>cagccagtccaccggcgactcttcgttagcgcttggcgtaaaatcggtttcagccggtgaccggg<br>ccattgcaatgggcgcctcatcctcagccagtggaagttattcaatggcaatgggcgtgtatgcca<br>attcgagcggcgcaaaatcggttgcgttaggttataaatctgtcgcgagcggagcaacttcctctg<br>cattaggttatcaagctactgcgagcggcgacgacagcgctgcttttggtaatggcgcaaaagcg<br>ataggcaccaactcagttgcccttggctcgggctctgtcgcccaggaagacaattccgtcgccgt<br>gggtaacagcaccactcagcggcagataacctacgttgctaaaggcgacatcaattccaccagt<br>accgatgccgttacaggtgcgcaaatttattctttaagtcaatccgtcgccgaccgactcggcgga<br>ggggcttccgttaatagtgatggtacagtgaatgcgcccctctacgaggtaggcacaggcatcta<br>caataacgtaggtagtgcattaagcgcacttaacacgtctatcactaacacagaggcctctgtcgc<br>aggattagccgaagacgcgctgttgtgggatgaaagcatcagcgcctttagcgctagc<br>green, *flag*×3; yellow, the gene of UreB349-363; blue, the gene of UreB158-172; red, the gene of linker (GGGGS)
Donor DNA for deletion of *ygeA* gene	gcgccacgacttagggctgacggagacgcttcatgccccggcaggaacaacccgtaccgagtt<br>actaacgctgaatgaagtgtgcgtactgccctgcgaccatccgcttgccgccaaaacggtactga<br>cgccagacgattttcaggggggaaaactttatcagcctctcccgcctggacagctatcgacagttgt

	tggatacgttatttgccgaacatcaggtgaagcgcagaatggtggtggaaacgcatagcgcggc<br>ctccgtctgcgcgatggtgcgcgccggggcgggcgtttcgatcgttaacccgctgaccgcgctg<br>gactatgccgccagcggcgtcaccgtccgccgcttcagcattgacgtgccctttaccgtcagcct<br>gattcgcccgctacaccgccctgcctccgcgctggttgacgccttcagtaagcatttacaaacgc<br>acctttcgcgcctggtcgaaccgctggaggttattctggggccgatgacgaaagcataaTgcgc<br>tctccttttgtaaagcattatggcagtgaagcaaacttacacatccgtttccagtctatttttcatcatg<br>ccggataacaggcgtcatccggcatgggaggggggattacacgccaatatttcttagcttctcgc<br>ccgccatcagcttgcgttcgatgtgctccagggtgacatttttggtttccggaatcagccagaaagt<br>gatgccgataaaagcgatattcagcgcggtgtagagccagaatgtacctgccgcgccaatgctg<br>tccaacagtgtcaggaatgtcgcgccgatgatcatgttcgatacccagtttgtcgtggttgaacag<br>gtaatgccaaaatcacggcatttcagcggctggatttccgaacacagtatccacaccaccggagc<br>ggcgctcatcgcgtaaccggcgatacacatcatcgtcatcccaacggaaagccaggagagacc<br>gcttgatgccgtaccgttatcaaactgcatcaggcagtagcccaacaccaatg
UreBm	NLSVGPATEALAGEGLIVTAGGIDTHIHFISPQQIPTAFASGVTT<br>MIGGGTGPADGTNATTITPGRRNLKWMLRAAEEYSMNLGFL<br>AKGNASNDASLADQIEAGAIGFKIHEDWGTTPSAINHALDVA<br>DKYDVQVAIHTDTLNEAGCVEDTMAAIAGRTMHTFHTEGAG<br>GGHAPDIIKVAGEHNILPASTNPTIPFTVNTEAEHMDMLMVC<br>HHLDKSIKEDVQFADSRIRPQTIAAEDTLHDMGIFSITSSDSQA<br>MGRVGEVITRTW
PAD4	FHYDRNNIAVGADESVVKEAHREVINSSTEGLLLNIDKDIRKI<br>LSGYIVEIEDTEGLKEVINDRYDMLNISSLRQDGKTFIDFKKY<br>NDKLPLYISNPNYKVNVYAVTKENTIINPSENGDTSTNGIKKIL<br>IFSKKGYEIG
mScarlet	MVSKGEAVIKEFMRFKVHMEGSMNGHEFEIEGEGEGRPYEG<br>TQTAKLKVTKGGPLPFSWDILSPQFMYGSRAFTKHPADIPDY<br>YKQSFPEGFKWERVMNFEDGGAVTVTQDTSLEDGTLIYKVK<br>LRGTNFPPDGPVMQKKTMGWEASTERLYPEDGVLKGDIKM<br>ALRLKDGGRYLADFKTTYKAKKPVQMPGAYNVDRKLDITSH<br>NEDYTVVEQYERSEGRHSTGGMDELYK
SadA<br>(green, signal peptite; yellow, A^{269} (The truncation point of $SadA^{269}$); gray, A^{644} (The truncation point of $SadA^{644}$); purple, A^{877} (The truncation point of $SadA^{877}$); blue, A^{1171}	MNRIFKVLWNAATGTFVVTSETAKSRGKKNGRRKLAVSALIG<br>LSSIMVSADALANAGNDTGDGVTPTGTQTGGKGWIAIGTDA<br>TANTYTNVDGASAAMGYKASAMGKWSTAIGSYSQSTGDSSL<br>ALGVKSVSAGDRAIAMGASSSASGSYSMAMGVYANSSGAKS<br>VALGYKSVASGATSSALGYQATASGDDSAAFGNGAKAIGTNS<br>VALGSGSVAQEDNSVAVGNSTTQRQITYVAKGDINSTSTDAVT<br>GAQIYSLSQSVADRLGGGASVNSDGTVNAPLYEVGTGIYNNV<br>GSALSALNTSITNTEASVAGLAEDALLWDESISAFSASHTGNA<br>SKITNLAAGTLAADSTDAVNGSQLFDTNEKVDKNTADIATNT<br>GSINQNTADITANTDSINQNTTDIAANTTSINQNTTDIATNTTNI<br>NSLSDSVTTLTDDALLWDAASGAFSAKHNGSDSKITNLAAGT<br>LAADSTDAVNGSQLFDTNEKVDQNTADITTNTNSINQNTTDI<br>ATNTTNINNLSDSITTLTDDALLWDAASGAFSANHNGSASKIT<br>NLAAGTLAADSTDAVNGSQLFATNENVSQNTADITTNTNSIN

(The truncation point of SadA^{1171}); red, A^{1292} (The truncation point of SadA^{1292}))	QNTTDIATNTTSINNLSDSITTLTDDALLWDAASGTFSASRSGS<br>ASKITNLAAGTLAADSTDAVNGSQLYETNQKVDQNTSAIADI<br>NTSITNLSSDNLSWNETTSSFSASHGSSTTNKITNVAAGELSEE<br>STDAVNGSQLFETNEKVDQNTTDIAANTTNITQNSTAIENLNT<br>SVSDINTSITGLTDNALLWDEDTGAFSANHGGSTSKITNVAAG<br>ALSEDSTDAVNGSQLYETNQKVDQNTSAIADINTSITNLGTDA<br>LSWDDEEGAFSASHGTSGTNKITNVAAGEIASDSTDAVNGSQ<br>LYETNMLISQYNESISQLAGDTSETYITENGTGVKYIRTNDNG<br>LEGQDAYATGNGATAVGYDAVASGAGSLALGQNSSSSIEGSIA<br>LGSGSTSNRAITTGIRETSATSDGVVIGYNTTDRELLGALSLGT<br>DGESYRQITNVADGSEAQDAVTVRQLQNAIGAVTTTPTKYYH<br>ANSTEEDSLAVGTDSLAMGAKTIVNADAGIGIGLNTLVMADA<br>INGIAIGSNARANHANSIAMGNGSQTTRGAQTDYTAYNMDTP<br>QNSVGEFSVGSEDGQRQITNVAAGSADTDAVNVGQLKVTDA<br>QVSRNTQSITNLNTQVSNLDTRVTNIENGIGDIVTTGSTKYFK<br>TNTDGADANAQGADSVAIGSGSIAAAENSVALGTNSVADEAN<br>TVSVGSSTQQRRITNVAAGVNNTDAVNVAQLKASEAGSVRY<br>ETNADGSVNYSVLNLGDGSGGTTRIGNVSAAVNDTDAVNYA<br>QLKRSVEEANTYTDQKMGEMNSKIKGVENKMSGGIASAMA<br>MAGLPQAYAPGANMTSIAGGTFNGESAVAIGVSMVSESGGW<br>VYKLQGTSNSQGDYSAAIGAGFQW*

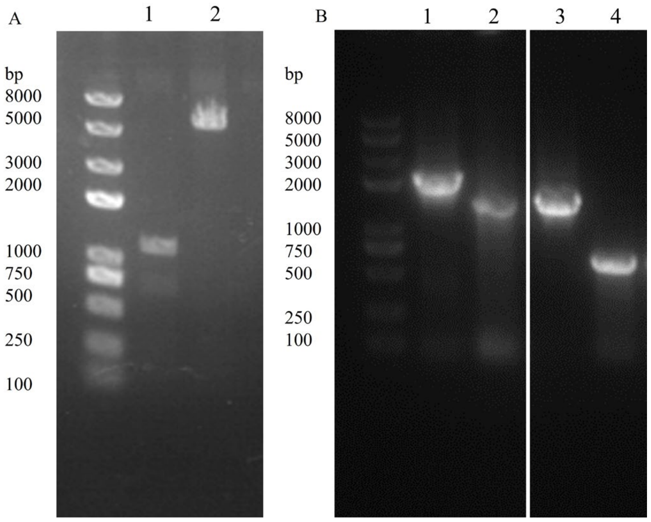

Figure S1. The *sadA*-deleted *S. typhimurium* and *ygeA*-*murI-double-deleted S. typhimurium* were confirmed by PCR. (A) PCR test for *sadA* gene of StmΔ*sadA* (lane1, 1274 bp) and wild-type strain (lane2, 5660 bp) using specific primers. (B) PCR test for *sadA* gene of StmΔ*ygeA*Δ*murI* strain and wild-type strain. Lane1, PCR product containing *ygeA* amplified from wild-type stain (2115 bp); lane2, PCR product containing *ygeA* amplified from StmΔ*ygeA*Δ*murI* (1407 bp); lane3, PCR product containing *murI* amplified from wild-type stain (1446 bp); lane4, PCR product containing *murI* amplified from StmΔ*ygeA*Δ*murI* (594 bp).

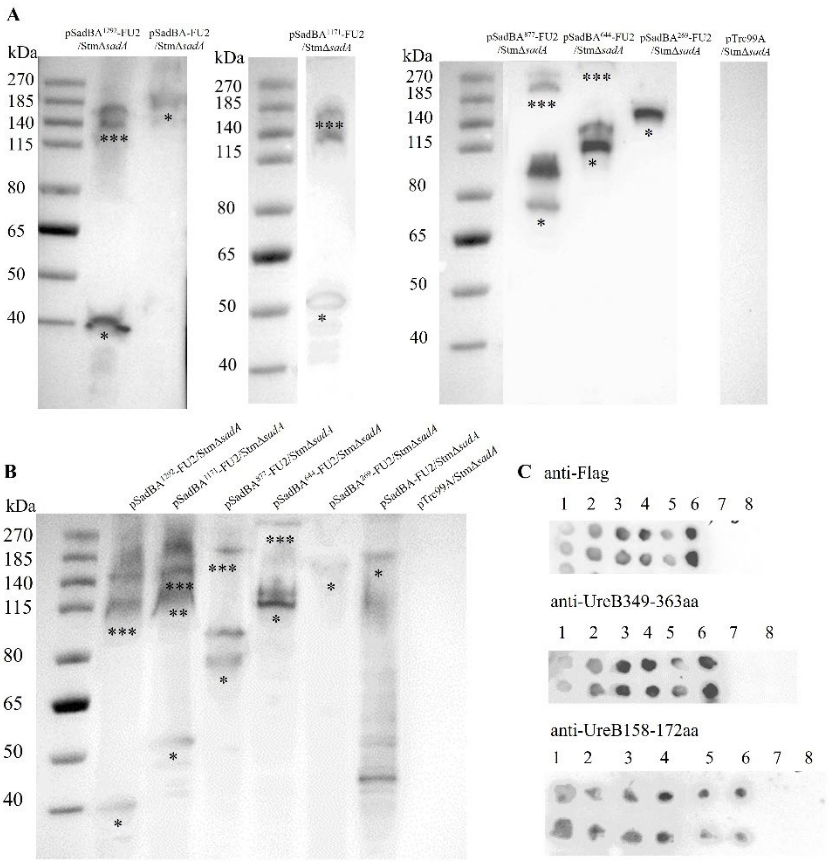

Figure S2. Western blot and dot blot of the displaying epitopes on the cell surface using full-length and truncated SadAs. Western blot showed that fused epitopes could be expressed in the Stm*ΔsadA* using the antibodies against UreB158–172aa (A1H10, A) and UreB349–363aa (A3C10, B). The putative positions of monomeric (*), dimeric (**) and trimeric (***) complexes was indicated under the bands. Dot blot of whole cells to analyze the surface-display of SadA derivatives on StmΔ*sadA* (C). Lane1, pSadBA^{1292}-FU2/StmΔ*sadA*; lane2, pSadBA^{1171}-FU2/StmΔ*sadA*; lane3, pSadBA^{877}-FU2/StmΔ*sadA*; lane4, pSadBA^{644}-FU2/StmΔ*sadA*; lane5, pSadBA^{269}-FU2/StmΔ*sadA*; lane6, pSadBA-FU2/StmΔ*sadA*; lane7, pTrc99A/StmΔ*sadA*; lane8, pFM/StmΔ*sadA*.

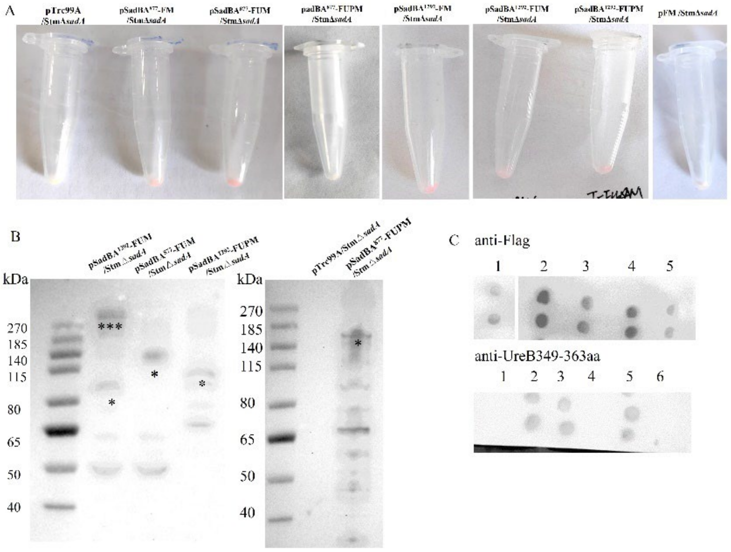

Figure S3. Confirmation of recombinant proteins displaying on the surface of cells using truncated SadAs as an anchoring motif. Compared with pTrc99A/StmΔ*sadA,* pSadBA^{1292}-FM/StmΔ*sadA,* pSadBA^{1292}-FUM/StmΔ*sadA,* pSadBA^{1292}-FUPM/StmΔ*sadA,* pSadBA^{877}-FM/StmΔ*sadA,* pSadBA^{877}-FUM/StmΔ*sadA,* pSadBA^{877}-FUPM/StmΔ*sadA* and pFM/Stm*ΔsadA* showed a pink color due the expression of mScarlet after induced with IPTG (A). Western blot showed that fused proteins could be expressed in the StmΔ*sadA* using the antibodies against UreB158–172aa(B). The putative positions of monomeric (*) and trimeric (***) complexes was indicated under the bands. Dot blot of whole cells to analyze display of recombinant proteins on Stm*ΔsadA* using A3C10 as the primary antibody (C). Lane1, pSadBA^{1292}-FM/StmΔ*sadA*; lane2, pSadBA^{1292}-FUM/StmΔ*sadA*; lane3, pSadBA^{1292}-FUPM/StmΔ*sadA*; lane4, pSadBA^{877}-FM/StmΔ*sadA*; lane5, pSadBA^{877}-FUM/StmΔ*sadA*; lane6, pTrc99A/StmΔ*sadA*.

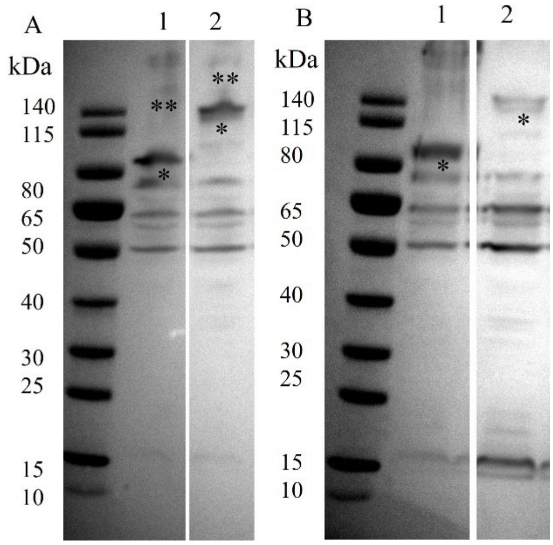

Figure S4. Western blot of heterologous proteins expressed in StmΔ*ygeA*Δ*murI* using the antibodies against Flag tag (A) and UreB158–172aa(B). Lane1, pSadBA^{1292}-FUM/ StmΔ*ygeA*Δ *murI* , lane2, pSadBA^{877}-FUM/StmΔ*ygeA*Δ*murI*. The putative positions of monomeric (*) and dimeric (**) complexes was indicated under the bands.

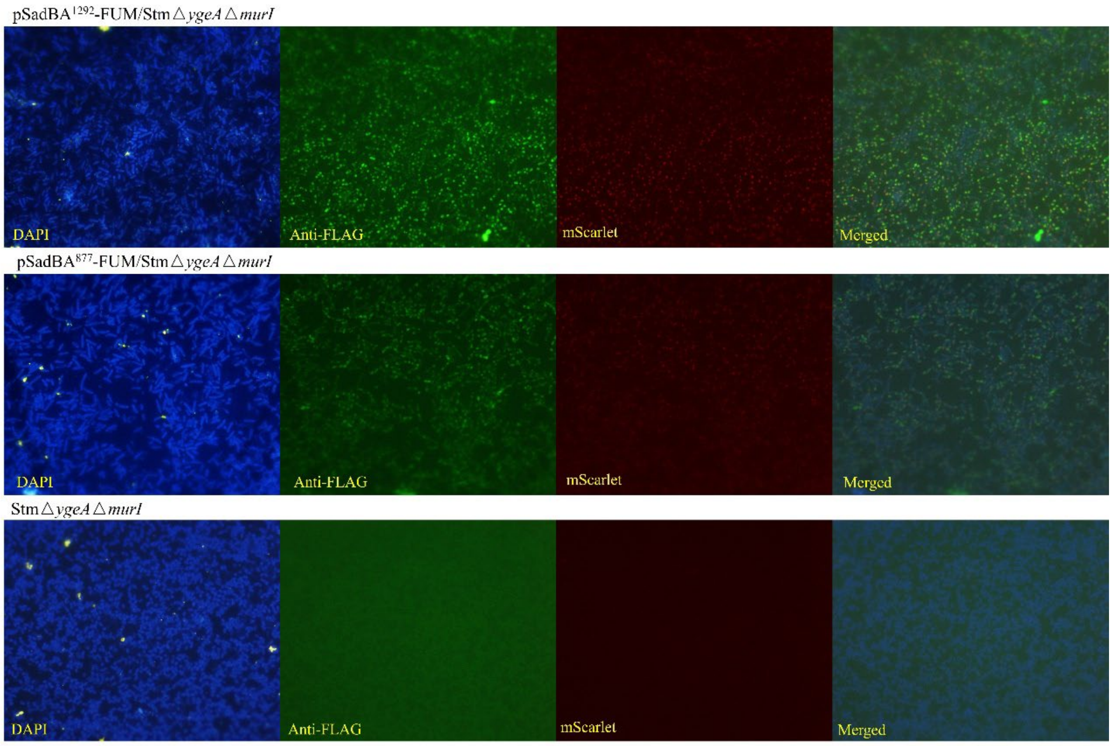

Figure S5. Cell surface display of Flag-tagged SadA derivatives on Stm△*ygeA*△*murI* by immunofluorescence staining using anti-Flag-tag primary antibody and Alexa Fluor 488 conjugated secondary antibody (Objective, 100×; Magnification, 1000×).
